# Benzodiazepine and Z-Drug Use and the Risk of Developing Dementia

**DOI:** 10.1093/ijnp/pyab073

**Published:** 2021-11-02

**Authors:** Francisco Torres-Bondia, Farida Dakterzada, Leonardo Galván, Miquel Buti, Gaston Besanson, Eric Grill, Roman Buil, Jordi de Batlle, Gerard Piñol-Ripoll

**Affiliations:** 1 Pharmacy Department, Clinical Neuroscience Research Group, IRBLleida, Arnau de Vilanova University Hospital, Lleida, Spain; 2 Unitat Trastorns Cognitius (Cognitive Disorders Unit), Clinical Neuroscience Research Group, Santa Maria University Hospital, IRBLleida, Lleida, Spain; 3 Pharmacy Department, Servei Català de la Salut (Catalan Health Services), Lleida, Spain; 4 Unitat d’Avaluació Clínica (Clinical Evaluation Unit), Institut Català de la Salut (Catalan Institute of Health), Lleida, Spain; 5 Accenture Innovation Center, Barcelona, Spain; 6 Barcelona Graduate School of Economics, Barcelona, Spain; 7 Universitat Oberta de Catalunya, Barcelona, Spain; 8 Group of Translational Research in Respiratory Medicine, Arnau de Vilanova University Hospital and Santa Maria University Hospital, IRBLleida, Lleida, Spain; 9 Biomedical Research Networking Center in Respiratory Diseases (Centro de Investigación Biomédica en Red de Enfermedades Respiratorias), Madrid, Spain

**Keywords:** Alzheimer’s disease, benzodiazepine, dementia, cohort study, cognitive decline, Z-drug

## Abstract

**Background:**

Benzodiazepines (BZDs) and Z-drugs (BZDRs) are among the most prescribed medications for anxiety and insomnia, especially among older adults. Our objective was to investigate the association between the use of BZDRs and the risk of dementia.

**Methods:**

A community-based retrospective cohort study was conducted based on the data available from 2002 to 2015 in Catalan Health Service. This cohort included all BZDR users (N = 83 138) and nonusers (N = 84 652) older than 45 years. A minimum 5-year lag window and an adjustment for psychiatric problems were applied for the data analysis.

**Results:**

The hazard ratio (HR) for the risk of incident dementia among BZDR users was 1.22 (95% CI = 1.15 to 1.31). This risk was not significant after adjusting the data confounding factors (HR = 1.01; 95% CI = 0.94 to 1.08). We observed a higher risk with short-to-intermediate half-life BZDs (HR = 1.11; 95% CI = 1.04 to 1.20) and Z-drugs (HR = 1.20; 95% CI = 1.07 to 1.33) than for intermediate-to-long half-life BZDs (HR = 1.01; 95% CI = 0.94 to 1.08). We demonstrated a higher risk of incident dementia (HR = 1.23; 95% CI = 1.07 to 1.41 and odds ratio = 1.38; 95% CI = 1.27 to 1.50, respectively) in patients who received 91 to 180 defined daily doses (DDDs) and >180 DDDs compared with patients who received <90 DDD. Regarding patient sex, the risk of dementia was higher in women than in men.

**Conclusion:**

We found that the incidence of dementia was not higher among all BZDR users. Short half-life BZDs and Z-drugs increased the risk of dementia at the highest doses, especially in female patients, showing a dose-response relationship.

**Significance Statement**

Previous studies suggest an association between benzodiazepine (BZD) and Z-drugs (BZDRs) consumption and increased risk of dementia and Alzheimer’s disease (AD) in particular, but this association is controversial. The current study analyzed the risk of BZDs and BZDRs to induce all types of dementia considering a lag window of 5 years between the drug consumption and the diagnosis of dementia in population from Mediterranean basin. We observed that BZDs users did not present an increased risk of dementia as a whole group. However, we observed an increased risk of dementia related with short-to-intermediate half-life BZD and BZDRs. This risk was higher in women and it increased with higher doses of BZD. These results address the importance of avoiding long-term use of these medications.

## Background

Benzodiazepines (BZDs) and their analogous Z-drugs (jointly referred to as BZDRs) are a class of psychoactive drugs widely used for generalized anxiety disorder and insomnia, especially in elderly people. BZDRs are also indicated for panic attacks, phobic disorders, obsessive-compulsive disorders, posttraumatic stress, epilepsy, and muscle spasms (Jackson et al., 2014). From a molecular point of view, BZDRs enhance the inhibitory effect of γ-aminobutyric acid at the γ-aminobutyric acid A subunit receptor by increasing the frequency of chlorine channel opening (Brunton and Hilal-Dandan, 2018). These drugs appear to act at the limbic, thalamic, and hypothalamic regions of the central nervous system, resulting in hypnotic, sedative, anxiolytic, and muscle-relaxant properties (Harvey, 2015).

BZDs are categorized into 3 groups according to their half-life: long (>24 hours), intermediate (6–24 hours), or short (<6 hours). Usually, short- and intermediate-acting BZDs are prescribed for insomnia, whereas longer-acting BZDs are reserved for anxiety (Hessmann et al, 2018). Although the maximum recommended length of treatment with BZDRs is between 2 and 4 weeks for insomnia or anxiety and, at most, 2 weeks for mixed anxiety-depressive disorders (MHRA, 2018), on many occasions, their usage becomes chronic. This long-term use of these medications has been demonstrated to increase the risk of falls and hip fractures and to have negative effects on cognition (Mura et al., 2013; Park et al., 2015). In fact, one of the concerns regarding the adverse effect of BZDRs in elderly people is the detrimental effect of these medications on cognition. In addition to the effects of chronic use of BZDRs, in geriatric patients, the half-lives of BZDs are extended because of age-related alterations in the pharmacokinetics and pharmacodynamics of the drugs, including changes in drug distribution and elimination, increasing the risk of adverse effects (Hessmann et al, 2018).

There are rational concerns regarding the use of these medications in elderly people and the increased risk of dementia and Alzheimer’s disease. At present, there is a lack of any solid biological mechanistic hypothesis to prove a causal link between BZDR use and dementia. Moreover, epidemiological evidence regarding the association between the use of BZDRs and the development of dementia is controversial. Some large epidemiological studies have shown that BZDR exposure increases the risk of developing dementia in elderly patients (Gomm et al., 2016; Shash et al., 2016; Penninkilampi et al., 2018). However, other studies did not find an association between the use of BZDs and the risk of dementia (Imfeld et al., 2015; Zhang et al., 2016; Biétry et al., 2017).

Because of these discrepancies in the results of previous studies, we decided to evaluate the association between the use of BZDRs and the incidence of dementia in a retrospective cohort of patients in the Sanitary Region of Lleida (SRL), Spain. We also analyzed our data for any relationship between the incidence of dementia and patient sex, BZD dose, and BZD half-life (short-to-intermediate or intermediate-to-long).

## METHODS

### Source of the Data

This study was a community-based retrospective cohort study carried out in 2016. The data about the population of BZDR users were provided in the Servei Català de la Salut (CatSalut) from January 1, 2002, to December 31, 2015. This health system provided health coverage to 358 070 inhabitants in the SRL in 2015, which represents 98% of the Lleida County population. Prescription data are part of the CatSalut medical prescription billing database. In addition to the active substance, each record contains information on the number of DDDs per year and accumulated dose at the end of the study period. CatSalut identifies each patient by means of their personal identification code (CIP). To ensure that anonymization in the database is provided, each individual was assigned a different sequential code in addition to the CIP. The assignment of the code to each CIP was blind for the study researchers and was available to only the 2 people who performed the data extraction and who did not participate in the design, statistical analysis, or discussion of the results. The control cohort and the clinical and demographic characteristics of each cohort were obtained from the data provided by the Institut Catala de la Salut. Population data are part of the primary care clinical station, which is the computerized medical history program used by all professionals in the Institut Catala de la Salut primary care network. The data were extracted using the Spoon computer application (Pentahoo Data Integration). For each patient, this spreadsheet contains birth date, situation (active, transferred, or dead), basic area of health (corresponding to a territory and its population that is attended by a primary care team mainly consisting of family physicians, pediatrics, nurses and administrative support staff), the selected diagnoses, and the diagnosis coding date.

#### Study Population

This cohort included all BZDR users older than 45 years who had a family physician registered in a basic area of health of SRL at the beginning of the study.

A minimum lag window of 5 years between the beginning of the consumption of BZDRs and the diagnosis of dementia was considered for the analysis to take into account the long latency of dementia development and to reduce the possibility of reverse causality. Due to this lag window period, all patients who started the consumption after December 31, 2010, were excluded from the analyses. Similarly, a 5-year period was excluded from the follow-up of controls to avoid immortal time bias.

Demographic information including age, sex, and comorbidities such as hypertension, diabetes mellitus, hyperlipidemia, stroke, myocardial infarction, depression, anxiety, other affective disorders, sleep disturbances, and insomnia were registered. The diagnosis of dementia was defined as case documentation with one of the following International Statistical Classification of Diseases and Related Health Problems 10th Edition codes: G30.0, G30.1, G30.8, G30.9, G31.0, G31.01, G31.83, G31.84, G31.85, F01.5, F01.50, F01.51, F02.8, F02.80, F02.81, F03.9, F03.90, or F03.91 (CIE-9MC 10ª revisión, 2016).

All patients who consumed any dose of BZDRs during the study period were included in the first database. From this database, (1) patients younger than 45 years at the beginning of the study, (2) patients with a diagnosis of dementia at the beginning of the study or during the first 5 years after the beginning of BZDR consumption, and (3) patients who passed away or changed their address to outside of the SRL during the period of the study were excluded.

According to these criteria, we detected 83 138 patients who received BZDR medication(s) between January 1, 2002, and December 31, 2015. We identified 84 652 patients older than 45 years who were never treated with BZDR medications during the same period of time; these patients were assigned as controls. The end of the observation period was defined as December 31, 2015 (end of the study), or the time when the diagnosis of dementia was confirmed.

### Exposure

BZDRs were categorized as N05BA (anxiolytics, benzodiazepine derivatives), N05CD (hypnotics and sedatives, benzodiazepine derivatives), and N05CF (hypnotics and sedatives, drugs related to BZDs, also called Z-drugs) according to the Anatomical Therapeutic Chemical classification system. All the aforementioned BZDRs have the approval of the Spanish Medicines Agency and therefore were included in the study (Agencia Española de Medicamentos y Productos Sanitarios, 2018).

According to the plasma half-life (t½), the BZDs were classified into 2 groups: short-to-intermediate half-life (t½ < 20 hours) and intermediate-to-long half-life (t½ > 20 hours). The use of BZDRs was defined as at least 1 prescription during the observation period, and BZDR use was evaluated based on the accumulated defined daily dose (DDD) for each patient throughout this period. The DDD is a technical unit of measurement that corresponds to the daily maintenance dose of a drug for its main indication in adults and for a given route of administration. The DDDs of active ingredients are established by the WHO and are published on the WHO Collaborating Center for Drug Statistics Methodology website (WHO, https://www.whocc.no/atc_ddd_index, 2018).

Based on the exposure amount, we divided the BZD users into 3 groups: (1) extremely low doses (≤90 DDDs), (2) low doses (91–180 DDDs), and (3) high doses (≥181 DDDs). Long-term consumption was defined as the use of ≥181 DDDs (Billioti et al., 2014).

### Statistical Analyses

Participants’ baseline characteristics are described by the number (%) or mean (SD), as appropriate. Cox proportional hazards regression models were used to estimate hazard ratios (HR) for the association between the consumption of BZDRs (ever/never consumption and consumed dose) and risk of dementia, taking age as the primary time variable. In addition, analyses according to DDD and patient sex were also performed. All models were adjusted by age, sex, hypertension, diabetes and dyslipidemia, and psychiatric problems such as depression, anxiety, and insomnia. Finally, to rule out any potential bias by age or sex, an exact matching on age and sex allowing the repetition of controls was performed. The matched database was then used to perform Cox proportional hazards regression models estimating HR for the association between the consumption of BZDRs (ever/never) and risk of dementia, taking age as the primary time variable. All analyses were performed using Tableau 2019.1 or Stata v12. The level of significance was fixed at .05.

## RESULTS

**Figure 1.**
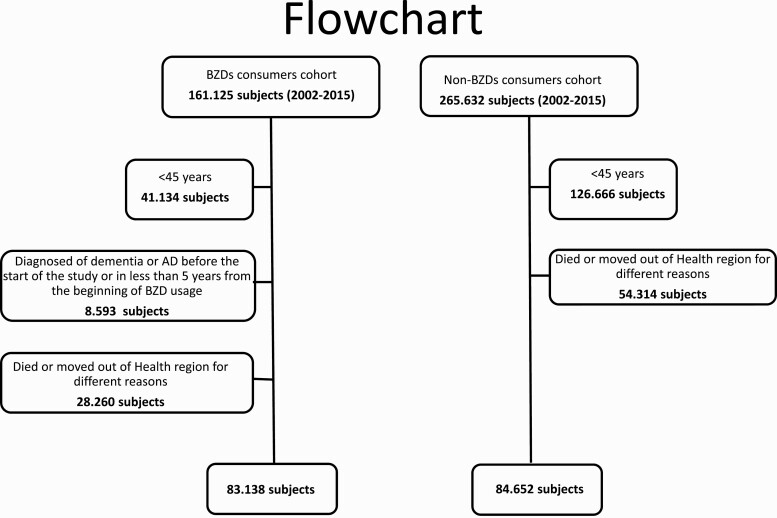
Flowchart of patients included in the analysis.

We identified 161.125 BZDR users from January 1, 2002, to December 31, 2015. From this population of BZDR users, 77.987 persons were excluded. Of those, 41.134 were excluded because they were younger than 45 years at the beginning of the study, 8.593 were excluded because they were diagnosed with dementia before the start of the study or within 5 years from the beginning of BZDR usage, and 28.260 were excluded because they died or moved out of the SRL for different reasons during the period of the study (Figure 1). Finally, 83 138 patients were included in the analysis. The control group consisted of 84 652 patients who had never used BZDRs. Demographic characteristics and the prevalence of comorbidities significantly differed between the 2 groups (Table 1). During the study period, we detected 4353 (5.2%) patients with dementia among the BZDR users and 1503 (1.8%) among the nonusers (*P* < .001) (Table 1).

**Table 1.** Characteristics of the Study Population According to the Consumption of BZDs and Z-Drugs

	Non-BZDs Users (n = 84.652)	BZDs Users (n = 83.138)	P
Age, mean (SD)	63.0 (12.5)	72.8 (14.6)	<.001
Women, n (%)	47 021 (55.6%)	50 064 (60.2%)	<.001
Hypertension, n (%)	29 832 (35.2%)	44 266 (53.2%)	<.001
Diabetes, n (%)	12 112 (14.3%)	18 162 (21.9%)	<.001
Dislipemia, n (%)	25 288 (29.9%)	33 187 (39.9%)	<.001
Cardiopathy, n (%)	3022 (3.6%)	6824 (8.2%)	<.001
Depression, n (%)	3443 (4.1%)	13 546 (16.3%)	<.001
Anxiety, n (%)	4856 (5.7%)	20 813 (25.0%)	<.001
Sleep disturbances, n (%)	1566 (1.9%)	2455 (3.0%)	<.001
Affective disorders, n (%)	698 (0.8%)	3118 (3.8%)	<.001
Dementia, n (%)	1503 (1.8%)	4353 (5.2%)	<.001

Abbreviations: BZD, benzodiazepine.

We observed a similar percentage of consumption for short-to-intermediate half-life BZDs and intermediate-to-long half-life BZDs: 73.7% and 73.4%, respectively. Z-drugs were used in just 15.3% of the study population. Regarding patient sex, women had used significantly more and higher doses (DDD) of BZDRs compared with men [1489.5 (2840.0) vs 967.6 (2405.8), *P* < .001]. These differences were observed in all subtypes of BZDs and Z-drugs (Table 2). In addition, we analyzed the number of BZDRs used among users with and without a diagnosis of dementia. We detected that among individuals without any type of dementia, 37.0% used only 1 type of BZDR during the period of the study, 30.7% used 2 types, 16.9% used 3 types, 8.4% used 4 types, and 6.9% used 5 or more types. Among patients with a diagnosis of dementia, 25.4% received 1 type of BZDR, 33.2% received 2 types, 19.9% received 3 types, 12.5% received 4 types, and 8.9% received 5 or more types.

**Table 2.** Characteristics of BZD and Z-Drug Consumption According to Patient Sex

	All	Women	Men	*P* ^*a*^
Total BZDs				
Users, n (%)	83 138 (100%)	50 064 (60.2%)	33 074 (39.8%)	<.001
Total DDD, mean (SD)	227.5 (2687.9)	1489.5 (2840.0)	967.6 (2405.8)	<.001
t1/2 short-intermediate				
Users, n (%)	61 265 (73.7%)	38 447 (46.3%)	22 788 (27.4%)	<.001
Total DDD, mean (SD)	1280.9 (2607.1)	1445.4 (2727.5)	1003.0 (2364.2)	<.001
t1/2 intermediate-long				
Users, n (%)	61 015 (73.4%)	37 537 (45.2%)	23 478 (28.2%)	<.001
Total DDD, mean (SD)	336.5 (1036.8)	665 (1208.8)	300.1 (1074.9)	<.001
Z-drugs				
Users, n (%)	12 731 (15.3%)	8224 (9.9%)	4507 (5.4%)	<.001
Total DDD, mean (SD)	594.5 (1130.6)	109.2 (548.4)	465.7 (958.7)	<.001

Abbreviations: BZD, benzodiazepine; DDD, defined daily dose.

^*a*^Comparison men-women.

The risk of incident dementia among BZDR users was evaluated by comparing the BZDR population with the control population. We found that the risk for dementia was higher among BZDR users (HR = 1.22; 95% CI = 1.15 to 1.31). This risk was not significant after adjustment of the data for age, sex, hypertension, diabetes, dyslipidemia anxiety, depression, and sleep disturbances (HR = 1.01; 95% CI = 0.94 to 1.08). Regarding the type of BZDs, after adjustment, we observed a higher risk for dementia with short-to-intermediate half-life BZDs (HR = 1.11; 95% CI = 1.04 to 1.20) and Z-drugs (HR = 1.20; 95% CI = 1.07 to 1.33) than for the intermediate-to-long half-life BZDs (HR = 1.01; 95% CI = 0.94 to 1.08) (Table 3). Cox proportional hazards models for BZDR consumption and risk of dementia in the sex- and age-matched database reported similar results (models stratified by matching pair id after exact matching by sex and age allowing for repetitions) (supplementary Table 1).

**Table 3.** Cox Proportional Hazards Models for BZD and Z-Drug Consumption and Risk of Dementia According to Subtypes of BZDs and Z-Drugs

	All population		Women		Men	
	Crude HR (95% CI)	Adjusted HR (95% CI)*	Crude HR (95% CI)	Adjusted HR (95% CI)*	Crude HR (95% CI)	Adjusted HR (95% CI)*
All BZDs						
Nonusers	ref	ref	ref	ref	ref	ref
BZDs users	1.22 (1.15 - 1.31)	1.01 (0.94 - 1.08)	1.49 (1.33 - 1.67)	1.28 (1.14 - 1.44)	1.09 (1 - 1.18)	1.09 (1.08 - 1.10)
Short-intermediate						
Nonusers	ref	ref	ref	ref	ref	ref
Short-intermediate half-life users	1.37 (1.28 - 1.47)	1.11 (1.04 - 1.20)	1.69 (1.5 - 1.91)	1.44 (1.27 - 1.63)	1.2 (1.11 - 1.31)	0.99 (0.91 - 1.08)
Intermediate-long						
Nonusers	ref	ref	ref	ref	ref	ref
Intermediate-long half-life users	1.24 (1.16 - 1.33)	1.01 (0.94 - 1.08)	1.48 (1.3 - 1.67)	1.26 (1.11 - 1.44)	1.11 (1.02 - 1.21)	0.9 (0.83 - 0.99)
Z-drugs						
Nonusers	ref	ref	ref	ref	ref	ref
Z-drugs users	1.60 (1.44 - 1.77)	1.20 (1.07 - 1.33)	1.86 (1.54 - 2.25)	1.46 (1.20 - 1.78)	1.42 (1.26 - 1.61)	1.09 (0.96 - 1.26)

Abbreviations: AbCI, confidence interval; BZD, benzodiazepine; CI, confidence interval; HR, hazard ratio.

*Adjusted by age, sex, hypertension, diabetes, dyslipemia, anxiety, depression, and sleep disturbances.

To examine the dose-response relationship, we analyzed the risk of dementia between groups divided by the exposure dose. We detected a dose-response relationship regarding the risk of dementia, because the patients who received 91 to 180 DDDs and >180 DDDs demonstrated a higher risk of incident dementia (HR = 1.26; 95% CI = 1.10 to 1.44 and HR = 1.49; 95% CI = 1.37 to 1.62, respectively) than those who received <90 DDDs (Table 4). This association remained significant after adjusting the data for confounding factors (HR = 1.23; 95% CI = 1.07 to 1.41 and HR = 1.38; 95% CI = 1.27 to 1.50, respectively) (Table 4).

**Table 4.** Cox Proportional Hazards Models for BZDR Consumption and Risk of Dementia According to Defined Daily Dose and Gender Groups

	All population		Women		Men	
	Unadjusted OR (95% CI)	Adjusted OR (95% CI)*	Unadjusted OR (95% CI)	Adjusted OR (95% CI)*	Unadjusted OR (95% CI)	Adjusted OR (95% CI)*
All BZDs						
<90 DDD	ref	ref	ref	ref	ref	ref
91–180 DDD	1.59 (1.39–1.82)	1.27 (1.11–1.47)	1.79 (1.47–2.20)	1.40 (1.14–1.73)	1.43 (1.19–1.72)	1.18 (0.98–1.43)
>180 DDD	2.24 (2.06–2.43)	1.52 (1.39–1.66)	2.09 (1.83–2.38)	1.56 (1.36–1.79)	2.24 (2.01–2.49)	1.49 (1.33–1.67)
Short-intermediate						
<90 DDD	ref	ref	ref	ref	ref	ref
91–180 DDD	1.41 (1.23–1.62)	1.23 (1.06–1.43)	1.34 (1.07–1.68)	1.18 (0.93–1.48)	1.45 (1.22–1.74)	1.27 (1.05–1.54)
>180 DDD	1.74 (1.60–1.90)	1.39 (1.27–1.52)	1.59 (1.38–1.83)	1.40 (1.21–1.62)	1.80 (1.61–2.00)	1.38 (1.23–1.56)
Intermediate-long						
<90 DDD	ref	ref	ref	ref	ref	ref
91–180 DDD	1.10 (0.95–1.29)	1.00 (0.85–1.18)	1.22 (0.93–1.60)	1.11 (0.84–1.46)	1.03 (0.85–1.24)	0.95 (0.78–1.16)
>180 DDD	1.57 (1.44–1.72)	1.30 (1.18–1.44)	1.53 (1.30–1.81)	1.40 (1.18–1.66)	1.54 (1.38–1.72)	1.26 (1.12–1.42)
Z-drugs						
<90 DDD	ref	ref	ref	ref	ref	ref
91–180 DDD	1.38 (1.07–1.78)	1.34 (1.03–1.76)	1.21 (0.78–1.88)	1.23 (0.78–1.96)	1.48 (1.09–2.02)	1.40 (1.00–1.95)
>180 DDD	1.17 (0.99–1.37)	1.04 (0.87–1.23)	1.15 (0.86–1.54)	1.15 (0.85–1.56)	1.15 (0.95–1.40)	0.98 (0.80–1.21)

Abbreviations: BZD, benzodiazepine; CI, confidence interval; DDD, defined daily dose; HR, hazard ratio; OR, odds ratio.

*Adjusted by age, sex, hypertension, diabetes, dyslipemia, anxiety, depression and sleep disturbances.

Regarding the type of BZDs, we observed a dose-response relationship among users of short-to-intermediate acting BZDs, because there was a consistently higher risk of dementia in patients who used 91 to 180 DDDs and >180 DDDs (HR = 1.21, 95% CI = 1.05 to 1.38 and OR = 1.28, 95% CI = 1.17 to 1.39, respectively) than with <90 DDDs. In the case of intermediate-to-long acting BZDs, there was a dose-response relationship only in doses higher than 180 DDDs (HR = 1.21; 95% CI = 1.10 to 1.32) (Table 4).

Regarding patient sex, the adjusted risk of dementia for all types of BZDs was higher in women (HR = 1.28; 95% CI = 1.14 to 1.44) than in men (HR = 1.09; 95% CI = 1.08 to 1.10). This risk was also higher in women for short-to-intermediate half-life BZDR (Table 3). By patient sex, the adjusted dose-response relationship risk was consistent and higher in women (HR = 1.32; 95% CI = 1.08 to 1.62 and HR = 1.44; 95% CI = 1.26 to 1.65, respectively) than in men (HR = 1.16; 95% CI = 0.97 to 1.39 and HR = 1.33; 95% CI = 1.19 to 1.48, respectively) (Table 4).

Given that the DDD used in our study was higher than that used in other studies, we evaluated the presence of a dose-response relationship not only according to the cutoff point used in previous studies (29) but also by dividing our study population into quartiles. The DDD values for the different quartiles were <173 DDDs for the first quartile, 173 to 960 DDDs for the second quartile, 961 to 2577 DDDs for the third quartile, and >2577 DDDs for the 4th quartile. Applying this analysis, the dose-response relationship was similarly replicated both for global BZD and subtypes of BZD and for the 2 subgroups of men and women (supplementary Table 2).

## Discussion

We evaluated the risk of BZDR use and the incidence of dementia in a community-based retrospective cohort. We included a 5-year lag window and adjusted the data by psychiatric covariates to overcome possible bias. Our results demonstrated an association between the use of BZDs and the incidence of dementia, especially in women and with high intakes of short half-life BZDRs.

Detecting risk factors for dementia is a fundamental step for its prevention. Normally, the presence of health problems increased in elderly patients, and as a result, polypharmacy is common among this population. BZDRs are a widely used medication for the treatment of anxiety and sleep disturbances, which are frequent health problems in geriatric patients. During recent years, several cohort studies have assessed the association between the long-term use of BZDRs and AD or dementia (López-Muñoz et al., 2011; Sonnenberg et al., 2012; Jackson et al., 2014; Billioti et al., 2015; Verger et al., 2018); however, other studies did not find this association (Imfeld et al., 2015; Zhang et al. 2016; Biétry et al., 2017).

There are some potential explanations for the discrepancies in the results of previous studies. Differences in sample size, follow-up time, covariate adjustment of the data or not, study design variation, and assessment of various degrees of cognitive impairment are some of the potential reasons the previous studies may have not led to a conclusive result. Another source of discrepancies could be the effect of perception bias or reverse causation bias that occurs when a pharmaceutical agent is prescribed for a prodromal manifestation of a disease that has not yet been diagnosed. These biases affect case-control and cohort studies, creating an artificial excess of cases in the group with prior use of the drug. Therefore, the association between the drug and disease risk is overestimated. The introduction of a lag time, the exclusion of patients with drug exposure for a defined period prior to diagnosis, and adjustment for potential psychiatric confounders can minimize perception bias in pharmacoepidemiological studies. In the present study, we included a 5-year lag time from the start of BZD consumption until the diagnosis of dementia and adjusted our data for several covariates, such as age, sex, hypertension, diabetes, and dyslipidemia, and some psychiatric disorders, such as depression, anxiety, and insomnia. Therefore, we believe that our results are minimally affected by perception bias. A similar lag time was also applied in other studies (Billioti et al., 2014; Gomm et al., 2016; Gray et al., 2016; Biétry et al., 2017; Tapiainen et al., 2018). In a recent meta-analysis of those studies that implemented the longest lag times of ≥5 years, there was a significant risk of dementia (OR = 1.30; 95% CI = 1.14 to 1.48) (Penninkilampi et al., 2018).

Regarding the type of BZDRs, we observed a statistically significant association between the consumption of short-to-intermediate BZDs and the risk of the development of dementia. This analysis was also carried out in several other studies. In the study by Billioti et al. (Billioti et al., 2015), an association was observed for both short-acting (OR = 1.43; 95% CI = 1.27 to 1.61) and long-acting BZDs (OR = 1.70; 95% CI = 1.46 to 1.98). Gomm et al. (Gomm et al., 2016) observed that the association between the use of BZDs and dementia was slightly stronger for long-acting BZDs than for short-acting BZDs (OR = 1.26; 95% CI = 1.15 to 1.39 and OR = 1.13; 95% CI 1.04 to 1.23, respectively) and increased with the dose, with similar results to ours. In the studies by Tapiainen et al. (Tapiainen et al., 2018) and Lee et al. (Lee et al., 2018), the authors also found an association between the use of BZDs and dementia in both short-acting and long-acting BZDRs.

He et al. (He et al., 2019) pooled 10 studies and observed that the effect of BZDs was associated with the use of BZDs with a longer half-life. However, another meta-analysis showed a significantly higher risk of dementia after consumption of short-acting BZDs (OR = 1.13; CI 95% = 1.02 to 1.26; *P* = .01). In contrast, this association was not observed with long-acting BZDRs (OR = 1.21; CI 95% = 0.99 to 1.49; *P* = .06) (Lucchetta et al., 2018).

The only study carried out in Spain, which included a 3-year lag period, showed a very weak increase in the risk of AD (OR = 1.05; 95% CI = 1.01 to 1.10) related to BZDRs, with no differences observed between short- or long-acting drugs (Aldaz et al., 2020).

Regarding Z-drugs, we observed that they increased the risk of dementia, especially in female patients and at the highest doses. Tapiainen et al. (Tapiainen et al., 2018) showed an increased risk of AD in patients taking Z-drugs (1.09; 95% CI = 1.05 to 1.12) after adjusting for another concomitant psychotropic medication. In a Taiwanese cohort, Chen et al. (Chen et al., 2012) also observed an effect on dementia related to Z-drugs.

We observed that the deleterious effect of BZDRs globally, especially short-half-life BZDRs, was greater in women than in men. Considering that BZD consumption in the global population is more prevalent in women, these data should be replicated in future studies and in other cohorts (Torres-Bondia et al., 2020). To our knowledge, no previous studies have shown this increased risk by patient sex. However, these results should be taken with caution because we cannot rule out a related survival bias. Thus, because women live longer and consume more BZD, this could explain a false increased risk of dementia in women and a lower risk in men.

This study has some strengths, including the long follow-up period, which allows us to analyze our data with a 5-year lag window between exposure and outcome and adjust our data for potential confounding factors to reduce the possibility of reverse causality bias. BZDRs dispensed by the pharmacies were used as a source of the data on drug use instead of drug prescriptions to avoid the problems with primary nonadherence. The Catalan health service is a public system that covers all citizens regardless of their socioeconomic situation; therefore, our study population can be considered a representative sample of the country’s population. Finally, taking into account the prolonged preclinical and prodromal period of some types of dementia, the long period of the study permitted us to have robust results related to the risk of dementia development.

Our study also had some limitations. The diagnosis of dementia was assessed based on the records in the claims data and was not verified. We considered a 5-year lag window as an inclusion criterion. This length of time can be considered short compared with the long preclinical and prodromal period of the disease; however, a longer lag window in our study was impossible because it would dramatically reduce the number of individuals included in the study. The data on the dispensing of medications from the Public Health System take into account the medications dispensed, but this does not guarantee the actual consumption by the patients. There were no data available on the specific clinical indications or the actual suitability of the use of BZDRs in our study population. Furthermore, in the database used for this study, there was no information available regarding some habits that may also act as potential covariates, such as alcoholism or smoking. A survival bias secondary to the design of the study cannot be excluded. Persons who died or moved out of health regions for different reasons were excluded from the study; the numbers are substantially high and a possible selection bias cannot be excluded. Another limitation of our study could be the consumption estimation through DDD because there may be discrepancies between the DDD values established by the WHO and the actual dose used in clinical practice. However, this technical unit of measurement permits the comparison of consumption data between countries. Another limitation is that concomitant antidepressant and antipsychotic treatments were not considered. In a recent study, there was an increased risk of developing AD with antidepressants and antipsychotics (Coupland et al., 2019). This could result in an overestimation of the risk of developing AD and other dementias from BZDRs in the present work. Finally, although the population included in the study was representative of the general population, it is not possible to guarantee that the prescribing habits of the family physicians were representative of the national prescribing habits of all primary care physicians.

In conclusion, we found that the incidence of dementia was not higher among all BZDR users. However, short half-life BZDR increased the risk of dementia at the highest doses, especially in female patients, showing a dose-response relationship. More studies will be necessary to confirm these results and to evaluate whether female sex increases the risk for the cognitive effects of BZDRs.

## Supplementary Material

pyab073_suppl_Supplementary_Material

## Data Availability

The data that support the findings of this study are available from the corresponding author on reasonable request.

## Notes

Interest Statement: None.
