# Supplementary material for: Benzodiazepine and Z-Drug Use and the Risk of Developing Dementia
Source: Int J Neuropsychopharmacol. 2021 Nov 2;25(4):261–8. doi: 10.1093/ijnp/pyab073 (PMC9017765; doi:10.1093/ijnp/pyab073)
Supplement: pyab073_suppl_Supplementary_Material [file pyab073_suppl_supplementary_material.docx]

Benzodiazepine use and the risk of dementia in Spanish population

Francisco Torres-Bondia^1*^, Farida Dakterzada^2*^, Leonardo Galván^3^, Miquel Buti^4^, Gaston Besanson^5,6^, Eric Grill^5^, Roman Buil^5,7^, Jordi de Batlle^8,9**^, Gerard Piñol-Ripoll^2**^

(1) Pharmacy Department, Clinical Neuroscience Research group, IRBLleida, Arnau de Vilanova University Hospital, Lleida, Spain.

(2) Unitat Trastorns Cognitius (Cognitive Disorders Unit), Clinical Neuroscience Research group, Santa Maria University Hospital, IRBLleida, Lleida, Spain.

(3) Pharmacy Department, Servei Català de la Salut (Catalan Health Services), Lleida, Spain.

(4) Unitat d'Avaluació Clínica (Clinical Evaluation Unit), Institut Català de la Salut (Catalan Institute of Health), Lleida, Spain.

(5) Accenture Innovation Center, Barcelona, Spain.

(6) Barcelona Graduate School of Economics (BGSE), Barcelona, Spain.

(7) Universitat Oberta de Catalunya (UOC), Barcelona, Spain

(8) Group of Translational Research in Respiratory Medicine, Arnau de Vilanova University Hospital and Santa Maria University Hospital, IRBLleida, Lleida, Spain.

(9) Biomedical Research Networking Center in Respiratory Diseases (Centro de Investigación Biomédica en Red de Enfermedades Respiratorias, CIBERES), Madrid, Spain.

* Co-first authors. FT-B and FD contributed equally to this study.

**Co-corresponding author

****Corresponding author:**

Gerard Piñol Ripoll

Cognitive Disorders Unit

Hospital Universitari Santa Maria

Rovira Roure nº 44, 25198, Lleida, Spain

Telephone: 34-937-727222 Ext. 173. Fax: 34-976-727366

E-mail: gerard_437302@hotmail.com

| **Table S1. Cox proportional hazards models for Benzodiazepines and z-drugs consumption and risk of dementia according to subtypes of BZDs and z-drugs, stratified by matching pair id (exact matching by sex and age allowing for repetitions)** | | | | | | |
| --- | --- | --- | --- | --- | --- | --- |
|  | All population | | Women | | Men | |
|  | Crude HR (95% CI) | Adjusted HR (95% CI)* | Crude HR (95% CI) | Adjusted HR (95% CI)* | Crude HR (95% CI) | Adjusted HR (95% CI)* |
| **All BZDs** | | | | | | |
| Non users | ref | ref | ref | ref | ref | ref |
| Users | 1.22 (1.16 - 1.29) | 1.01 (0.95 - 1.06) | 1.37 (1.25 - 1.51) | 1.19 (1.08 - 1.31) | 1.16 (1.09 - 1.23) | 0.93 (0.87 - 0.99) |
| **Short-intermediate half-life** | | | | | | |
| Non users | ref | ref | ref | ref | ref | ref |
| Users | 1.33 (1.26 - 1.41) | 1.05 (0.99 - 1.11) | 1.49 (1.34 - 1.65) | 1.25 (1.12 - 1.38) | 1.26 (1.18 - 1.35) | 0.97 (0.9 - 1.04) |
| **Intermediate-long half-life** | | | | | | |
| Non users | ref | ref | ref | ref | ref | ref |
| Users | 1.21 (1.14 - 1.28) | 0.93 (0.88 - 0.99) | 1.33 (1.19 - 1.48) | 1.09 (0.98 - 1.22) | 1.15 (1.07 - 1.23) | 0.87 (0.81 - 0.93) |
| **z-drugs** | | | | | | |
| Non users | ref | ref | ref | ref | ref | ref |
| Users | 1.38 (1.25 - 1.51) | 0.94 (0.85 - 1.03) | 1.48 (1.24 - 1.76) | 1.09 (0.91 - 1.3) | 1.32 (1.18 - 1.48) | 0.88 (0.78 - 0.99) |
| *Adjusted by age, sex, hypertension, diabetes, dyslipidaemia, anxiety, depression and sleep disturbances. | | | | | | |

**Table S2. BZD consumption and risk of disease dementia according to defined daily dose (quartiles) and gender groups.**
